# Comparison between Speckle Plethysmography and Photoplethysmography during Cold Pressor Test Referenced to Finger Arterial Pressure

**DOI:** 10.3390/s23115016

**Published:** 2023-05-24

**Authors:** Jorge Herranz Olazabal, Ilde Lorato, Jesse Kling, Marc Verhoeven, Fokko Wieringa, Chris Van Hoof, Willem Verkruijsse, Evelien Hermeling

**Affiliations:** 1 IMEC NL, 5656 AE Eindhoven, The Netherlands; 2 Faculty of Engineering Science, Katholieke Universiteit Leuven (KUL), 3000 Leuven, Belgium; 3 Division of Internal Medicine, Department of Nephrology, University Medical Center Utrecht, 3584 CX Utrecht, The Netherlands; 4 IMEC, 3000 Leuven, Belgium; 5 Philips Research, 5656 AE Eindhoven, The Netherlands

**Keywords:** speckle contrast, dual wavelength, SPG, PPG, CPT, low perfusion

## Abstract

Speckle Plethysmography (SPG) and Photoplethysmography (PPG) are different biophotonics technologies that allow for measurement of haemodynamics. As the difference between SPG and PPG under low perfusion conditions is not fully understood, a Cold Pressor Test (CPT—60 s full hand immersion in ice water), was used to modulate blood pressure and peripheral circulation. A custom-built setup simultaneously derived SPG and PPG from the same video streams at two wavelengths (639 nm and 850 nm). SPG and PPG were measured at the right index finger location before and during the CPT using finger Arterial Pressure (fiAP) as a reference. The effect of the CPT on the Alternating Component amplitude (AC) and Signal-to-Noise Ratio (SNR) of dual-wavelength SPG and PPG signals was analysed across participants. Furthermore, waveform differences between SPG, PPG, and fiAP based on frequency harmonic ratios were analysed for each subject (n = 10). Both PPG and SPG at 850 nm show a significant reduction during the CPT in both AC and SNR. However, SPG showed significantly higher and more stable SNR than PPG in both study phases. Harmonic ratios were found substantially higher in SPG than PPG. Therefore, in low perfusion conditions, SPG seems to offer a more robust pulse wave monitoring with higher harmonic ratios than PPG.

## 1. Introduction

Speckle Plethysmography (SPG) and Photoplethysmography (PPG) are fundamentally different biophotonics methods, both based on the measurement of photons to analyse blood dynamics in living tissue. SPG is a technology based on the analysis of Laser Speckle Contrast Imaging (LSCI) [1,2,3,4,5,6]. Spatial changes in speckle contrast are calculated from a sequence of video frames, generating a single value per frame. Changes in speckle contrast are produced by variations, such as movement of the target under analysis (in this case, human tissue cells: skin, blood vessels, blood cells, etc.).

PPG is a technology based on the measurement of light intensity. This technology is used to estimate modulations in the absorption of light by a living tissue, which relates to blood volume [7]. When cameras are used, intensity changes are calculated throughout a sequence of video frames producing an average value per frame.

Simultaneous monitoring of SPG and PPG could be used to study changes induced by blood circulation modulations [8]. A well-known technique used to systemically manipulate the peripheral circulation is the Cold Pressor Test (CPT) [9], which is performed through the application of cold temperatures to a part of the body. To preserve core temperature, sensory nerves trigger a systemic reaction, known as cold-induced vasoconstriction, leading to a decrease in the vessel diameter and increase in tone of the vessel walls. This results in elevated blood pressure (BP), an increase in Pulse Wave Velocity (PWV), and reduced skin blood flow [10]. Pulse Pressure (PP), which is calculated as the difference between systolic and diastolic BPs, has also been proven to increase [11]. Stroke volume decreases, which in turn decreases PPG amplitude [11]. For PPG, this decrease in stroke volume deteriorates the Signal-to-Noise Ratio (SNR) and pulse strength, which sometimes even results in a complete loss of the signal [12]. Different parts of the body react differently to the CPT. While core body parts do not seem affected, peripheral parts typically react with a decrease in the AC-amplitude of PPG. This has been demonstrated for ear and finger measurements [13].

The effects of the CPT on peripheral Near-Infrared SPG have been explored by Ghijsen et al. [1], whose findings show an increase in the time delay between SPG and PPG. SNR changes were also observed but not extensively analysed. Nevertheless, these observations suggested that SPG might be more robust to changes under low perfusion conditions compared to PPG. Therefore, SPG might become a promising alternative to monitor peripheral circulation under low perfusion conditions [11,12], although it deserves further investigation.

To our knowledge, the SNR and morphology of dual-wavelength SPG and PPG during the CPT have never been investigated before. This investigation focusses on analysing the changes induced by a lowered perfusion condition on SPG and PPG. The experiments were performed using a custom setup, which can measure simultaneous SPG and PPG from skin at two wavelengths (639 nm and 850 nm) multiplexed in time. Dual-wavelength SPG and PPG were measured at the right index finger location before and during the CPT.

The change produced by the CPT in AC-amplitude of dual-wavelength SPG and PPG signals across 10 subjects was evaluated. In addition, changes in SNR due to the CPT were calculated for SPG and PPG at two wavelengths. Furthermore, using frequency harmonic ratios, SPG and PPG waveform morphology characteristics were analysed for all subjects and compared to the finger Arterial Pressure (fiAP) reference.

## 2. Materials and Methods

The custom setup used for this investigation incorporated a miniature CMOS camera, and two laser diodes with wavelengths of 639 nm (Thorlabs HL6358MG, Thorlabs, Newton, NJ, USA, 10 mW) and 850 nm (Thorlabs L850P010, 10 mW), embedded in a finger-clip. These wavelengths were chosen because of their difference in penetration depth within human tissue. This, in turn, is caused by the different absorption and scattering coefficient of tissue components at these wavelengths. Especially, the spectral absorption differences between reduced and oxidized haemoglobin have been thoroughly exploited for oxygen saturation measurement (pulse oximetry, based on PPG). Even if absorption is not expected to have a significant effect on SPG performance [2], the SPG waveform still may be affected by the difference in penetration depth. The setup was configured to allow for the simultaneous measurement of reflective SPG and PPG multiplexing in time between the two wavelengths. The raw camera video stream was recorded at a framerate of 80 Frames Per Second (FPS), while the 639 nm and 850 nm light sources were alternating. We then separated the even and uneven frames into two video streams of 40 FPS each with only one wavelength. The distance between the camera and the finger skin was approximately 1 cm. The subjects positioned their right index finger on the setup.

A Finapres Nova (Finapres Medical Systems, Enschede, The Netherlands) was used as a reference device to monitor fiAP, based on the Peñáz method [14]. The custom experimental system was synchronized with the reference device using the acquisition platform BIOPAC MP160 (BIOPAC Systems Inc., Goleta, CA, USA). The Finapres finger cuff was applied on the right middle finger. This device was used as reference for heart rate and for harmonic ratios comparison.

The experimental study was performed on 10 subjects without known medical conditions. Both the custom setup and the Finapres recorded continuously during the protocol. The study consisted of two experimental phases: baseline and CPT. During the baseline phase, which lasted 1 min, the subject was asked to sit relatively still. Whereas, during the CPT, the subject was asked to submerge the left hand in ice water (total hand immersion) for 1 min while the reference and custom setup were recording the signals on the right-hand fingers. The duration of 1 min was adapted following the previous literature [15,16]. No metabolic activities were considered for this study. The ice water was prepared by mixing 3 litres of cold tap water with 0.5 kg of ice cubes, to be used after waiting for 5 min at room temperature. The experiment took place in a laboratory with an ambient temperature of 20 (+/−2) degrees Celsius.

**Figure 1:**
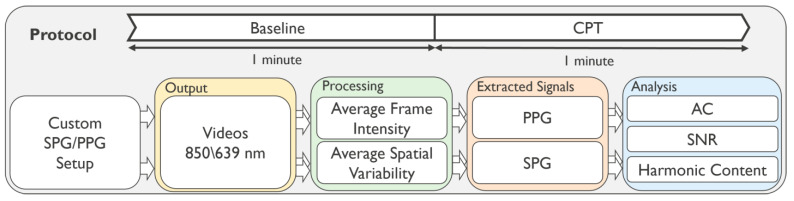
Flow diagram of the experimental protocol and data analysis of the experiments at baseline and CPT phases.

The subject population consisted of 10 healthy participants ranging from 28 to 53 years old; all subjects were Caucasian, with a 40 percent female population. The experimental protocol was evaluated and executed in accordance with the Declaration of Helsinki 2008 (protocol number: IM-NL-STUDY-2022-0029), and all subjects signed the informed consent prior to the study. Figure 1 summarizes the experimental protocol and data analysis pipeline.

### 2.1. Analysis

**Figure 2:**
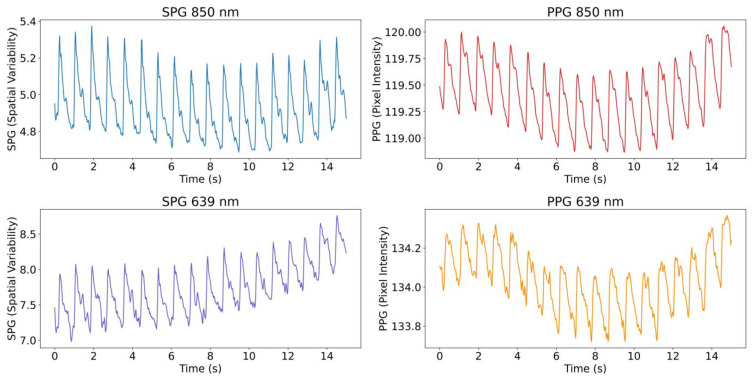
Example from Subject 1 of a 15 s period of the 4 simultaneous signals extracted from the videos. The signals shown were low pass filtered (10 Hz) and inverted. Note the quasiperiodic nature of SPG and PPG. The fast pulse waves originate from the heartbeat (arterial component dominates), the slower baseline variations mainly originate from respiration (venous component dominates).

All the processing and analysis was performed using Python programming language (Python Software Foundation 3.8). Raw videos were recorded at 80 FPS of the index finger, while using synchronized illumination at two alternating wavelengths (40 FPS each). After separating the alternating frames by wavelength, SPG and PPG were extracted for each wavelength at 40 samples per second. The equations used to compute SPG and PPG [17] are shown below. Let $I\left(t\right)$ be a frame of one of the two video streams at time t, with resolution N×M, corresponding to 640×400 pixels. The *PPG* was extracted by calculating the average pixel intensity of the frame as

(1) $$PPG\left(t\right)=\frac{1}{NM}\sum_{n=0}^{N-1}\sum_{m=0}^{M-1}I_{n,m}\left(t\right),$$

where n and m represent the two indices running through the two frames’ dimensions. *SPG* was calculated from the same video as the average spatial variability of each frame as

(2) $$SPG\left(t\right)=\frac{1}{NM}\sum_{n=0}^{N-1}\sum_{m=0}^{M-1}{\sigma \left(t\right)}_{n,m},$$

with ${\sigma \left(t\right)}_{n,m}$ being the spatial variability matrix calculated in the neighbourhood of the pixel at position n,m. Following the well-known standard deviation definition, the spatial variability can be expressed as

(3) $${\sigma \left(t\right)}_{n,m}=\sqrt{\left(\frac{1}{{\left(K+1\right)}^{2}}\sum_{x=n-\frac{K}{2}}^{n+\frac{K}{2}}\sum_{y=m-\frac{K}{2}}^{m+\frac{K}{2}}{I_{x,y}\left(t\right)}^{2}\right)-{\left(\frac{1}{{\left(K+1\right)}^{2}}\sum_{\hat{x}=n-\frac{K}{2}}^{n+\frac{K}{2}}\sum_{\hat{y}=m-\frac{K}{2}}^{m+\frac{K}{2}}I_{\hat{x},\hat{y}}\left(t\right)\right)}^{2}},$$

with K+1 being the kernel size, here equal to 7 pixels based on the literature [18]. An example of the extracted signals from the videos, both SPG and PPG at the two wavelengths, is shown in Figure 2.

#### 2.1.1. AC-Amplitude and SNR Calculations

PPG and SPG, obtained from the videos at both wavelengths, were further analysed to monitor the changes in AC and SNR between the baseline and CPT phases. The reference signal, fiAP, was included in the AC analysis; the AC of a BP signal can also be indicated as PP. The reference was not included in the SNR analysis since the signal is not raw but prefiltered by the Finapres system. However, fiAP was used for the estimation of the fundamental frequency in the SNR estimation.

**Figure 3:**
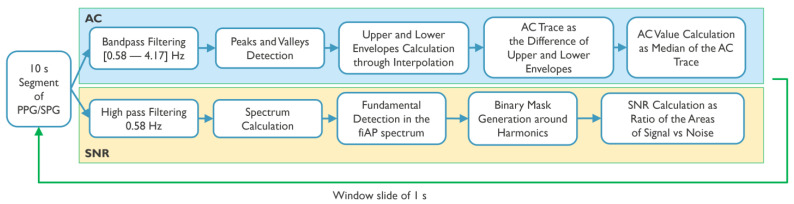
Processing pipeline for AC and SNR calculation.

Both AC-amplitude and SNR calculations were performed using a sliding window approach, with a window size of 10 s and an overlap of 9 s. The window size was chosen taking into account the trade-off between time and frequency resolutions. Both phases were included in this calculation. The two phases were separated, and 5 s signals were removed at the start and end of the baseline and CPT phases in order to avoid motion artifacts that could be present, e.g., due to the subject submerging the left hand in the ice water. Figure 3 summarizes the processing steps for the calculation of AC-amplitude and SNR.

For the AC-amplitude calculations, each window of the signals of interest (SPG, PPG, and fiAP) in both phases and wavelengths was first filtered using a bandpass filter (Butterworth cut-off frequencies: 0.58 Hz–4.17 Hz). Afterwards, peaks and valleys were located, and an interpolation was performed to obtain a line connecting all the peaks and a line connecting all the valleys, similar to an upper and lower envelope. The AC-amplitude of the signal in the current window was then calculated as the median value of the difference between the upper and lower envelopes.

**Figure 4:**
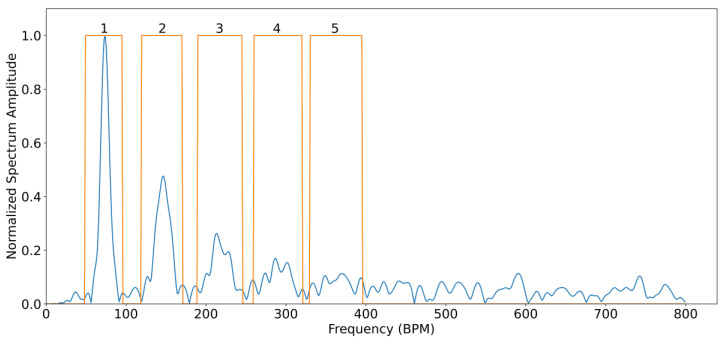
Typical spectrum of SPG from a single 10 s window in blue, with superimposed binary spectral mask (orange) describing bands containing signal (“1”) and noise (“0”). Numbering of the binary spectral mask refers to: 1—Fundamental, 2—Second harmonic, 3—Third harmonic, 4—Fourth harmonic, 5—Fifth harmonic.

For all subjects, the SNR was analysed by comparing the spectral power contained between certain frequency bands considered as signal and frequency bands of noise. This is carried out using fiAP as reference for the fundamental frequency peak, similar to the method described by Gerard de Haan et al. [17]. A binary mask was created to split the spectrum between signal and noise. This binary mask was equal to “1” in the frequency bands containing the fundamental frequency peak, calculated from fiAP reference, and in the 4 following harmonics of SPG and PPG. The rest of the binary mask was equal to “0”, representing frequencies corresponding to noise. The spectrum, limited to 800 beats per minute (BPM) was calculated, using zero-padding and a Hanning window, on the high passed filtered signals with a cut-off frequency of 0.58 Hz, in order to remove possible baseline and respiratory contributions. The width of the fundamental band was empirically set to 20 frequency bins and proportionally increased for the harmonic bands [17]. An example of this procedure is visible in Figure 4. More in details, let Y(f) be the spectrum of the current 10 s window of the signal of interest (SPG, PPG), and U(f) the described binary mask; the *SNR* can be calculated as

(4) $$SNR\left[dB\right]=10\;\log_{10}\left(\frac{\sum_{f=0}^{800\;BPM}U\left(f\right){Y(f)}^{2}}{\sum_{f=0}^{800\;BPM}(1-U\left(f\right)){Y(f)}^{2}}\right)$$

#### 2.1.2. Harmonic Ratio Calculation

The harmonic content of the signals was analysed to quantify the morphology differences between SPG, PPG, and fiAP. This analysis was based on the ratio between the different frequency harmonics of the signal and the fundamental, inspired by Ghijsen et al. who defined the Third Harmonic Ratio (THR) [1]. In this work, Second Harmonic Ratio (SHR) and Fourth Harmonic Ratio (FHR) were introduced and included in the analysis together with THR. The harmonics of a signal are influenced by its morphology, and the use of the three harmonic ratios allows for better comparison of the morphological differences and similarities between PPG, SPG, and fiAP. In all three cases, harmonic ratios are calculated as the ratio between the amplitude of the pertaining harmonic peak of interest and the fundamental peak.

**Figure 5:**
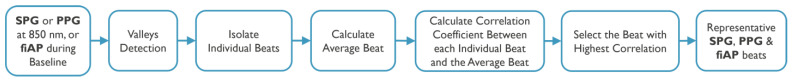
Processing pipeline for the selection of the representative beat.

The signals chosen for this analysis were produced by the 850 nm laser diode during the baseline test phase since they show the highest quality across subjects. The harmonic content was analysed on a representative beat per signal. This representative beat was selected by first calculating an average beat of each signal per subject and then selecting the real pulse that correlated the most with the average beats. A summary of the steps can be found in Figure 5.

**Figure 6:**
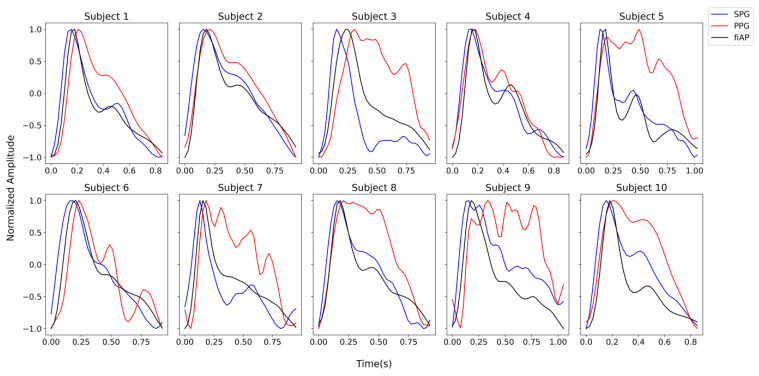
Representative SPG, PPG, and fiAP beats per subject, measured during the baseline test phase. The illumination chosen for this analysis was a reflective 850 nm laser diode. The representative beats are not simultaneous between the different signals.

The representative beats are concatenated 10 times with the purpose of obtaining a Fourier frequency spectrum with reasonable resolution. This results in a frequency spectrum for individual PPG, SPG, and fiAP beats. Figure 6 shows the representative beat of the collected signals for all the subjects during the baseline phase. It should be noted that the representative beats may not be the same, timewise, for all the three signals because the representative beats are selected based on the correlation coefficient.

#### 2.1.3. Statistical Analysis

For each subject, phase, signal, and wavelength a median value is calculated for both AC and SNR. These values are then analysed with paired comparisons. Four statistical comparisons were performed to analyse the significance of the changes. Two main statistical tests were used, assuming the populations have significant deviations from normality. Firstly, the Wilcoxon signed-rank test was chosen as a non-parametric version of the paired *t*-test, in order to perform paired comparisons. Secondly, the Levene’s test was chosen to compare variances. Four comparisons were made:

- Comparison between phases of the study (baseline and CPT): the Wilcoxon signed-rank test was used inter-subject to analyse if the changes in both AC-amplitude and SNR between the baseline and CPT phases were significant for the signals of interest. This comparison was carried out since it is expected for PPG to have a reduction in AC-amplitude during the CPT and consequently SNR [15], but it is not clear whether SPG will react in the same way.
- Comparison between experimental signals (SPG and PPG): the Wilcoxon signed-rank test was used to test if SNR SPG was significantly higher than SNR PPG for both study phases and wavelengths. This comparison was performed because it has been suggested that SPG has a higher SNR than PPG during the CPT [1].
- Comparison between SNR variance of experimental signals (SPG and PPG): the Levene’s test was used inter-subject to analyse if the SNR variance was significantly different between SPG and PPG. For this test all the SNR estimations were used as populations, and not the median, as for the other tests. This comparison was performed because we expected the SNR of SPG to be more consistent across subjects compared to PPG.
- Comparison between harmonic ratios of the signals (fiAP, SPG, and PPG): the Wilcoxon signed-rank test was used inter-subject to test if SHR, THR, and FHR were significantly higher between the different pairs of signals. These comparisons were chosen to verify whether the harmonic content of SPG is higher compared to PPG, and more similar to fiAP.
- In addition, the Pearson’s correlation coefficient and the regression line were estimated for the three harmonic ratios to compare fiAP morphology with SPG and PPG.

## 3. Results

### 3.1. AC-Amplitude Results

**Figure 7:**
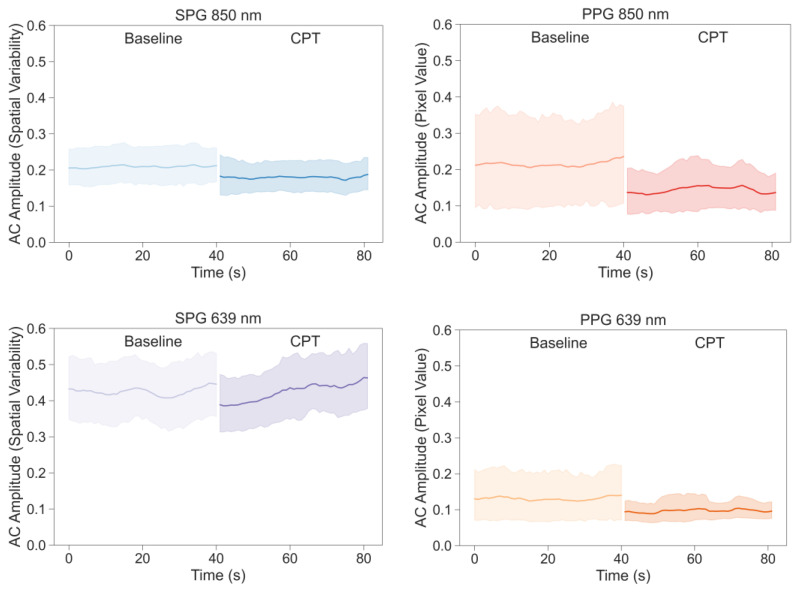
Inter-subject average values and 95% CI of AC-amplitude of the signals extracted from the videos during baseline and CPT phases.

**Figure 8:**
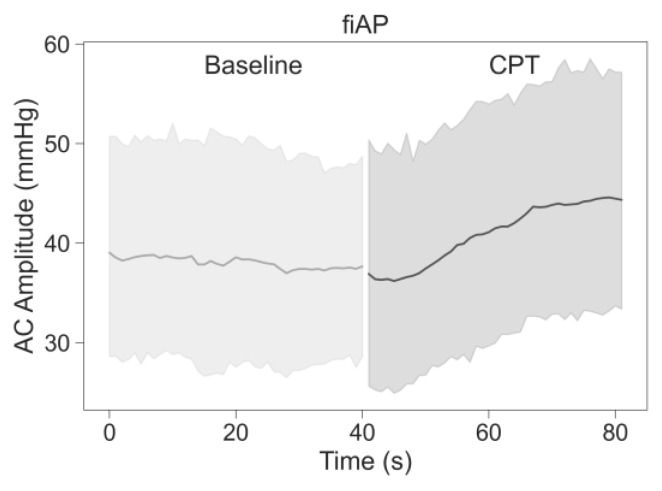
Inter-subject average values and 95% CI of AC-amplitude of the fiAP signals during baseline and CPT phases.

AC-amplitude was analysed for the experimental signals, SPG and PPG at 850 nm and 639 nm, as well as for fiAP. Figure 7 and Figure 8 show the inter-subject average and 95% Confidence Interval (CI) of AC-amplitude of each signal calculated independently for both phases of the study (baseline and CPT). On average, all signals show a reduction in AC-amplitude during the CPT; SPG at 639 nm shows a positive slope during the CPT, whereas the AC-amplitude of fiAP shows an increase in the CPT phase, as expected.

**Figure 9:**
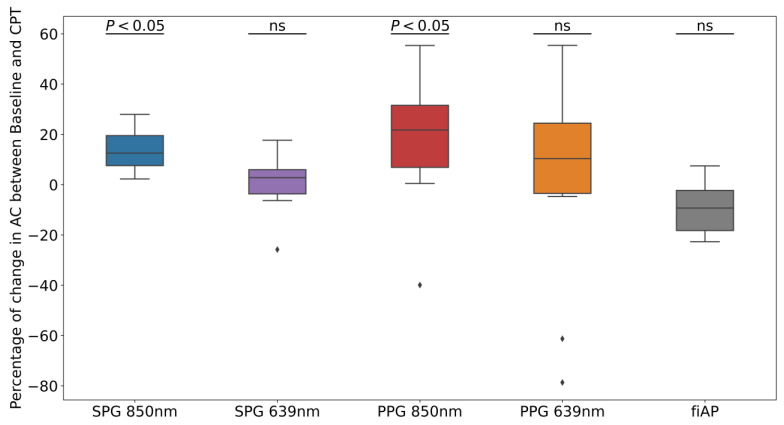
Percentage of AC-amplitude change between baseline and CPT on each of the experimental and reference signals for all subjects. *p*-values from Wilcoxon test prove the significance of the rejection of the null-hypothesis, indicating that AC-amplitude is significantly higher at baseline than at CPT phase at 850 nm. The term “ns” refers to non-significant.

To analyse if the reduction in AC-amplitude was significant, a statistical analysis was performed. Figure 9 shows the percentage of AC-amplitude change between baseline and CPT on each of the signals extracted from the videos and the reference fiAP for all subjects.

The change in SPG AC-amplitude (average ± standard deviation) was 14% ± 8% and 1% ± 11% for 850 nm and 639 nm, respectively, whereas for PPG, it was 18% ± 25% for 850 nm, and 3% ± 41% for 639 nm. The fiAP reference showed changes of −9% ± 10%. The positive average change indicates that the AC-amplitude was higher during baseline than during the CPT, whereas the negative average change indicates a higher AC-amplitude during the CPT.

The results of the Wilcoxon signed-rank test are shown in Figure 9 on each boxplot. The AC-amplitude during baseline is significantly higher than the one during the CPT for SPG and PPG at 850 nm, and not significant for the signals at 639 nm. The change is also not significant for fiAP, which is expected to react to the CPT with an increase in PP.

### 3.2. SNR Results

**Figure 10:**
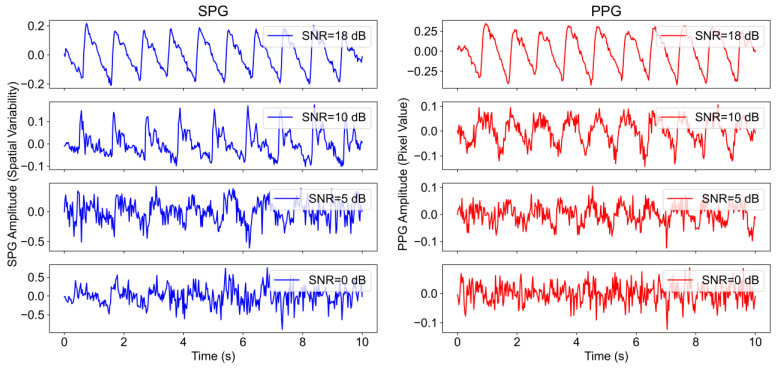
Example of SPG and PPG signals at different quality levels. From top to bottom: 18 dB, 10 dB, 5 dB, and 0 dB.

Example signals of both PPG and SPG with different SNR levels are shown in Figure 10. More concretely, four different quality levels ranging from 18 dB to 0 dB, depicting the different quality levels indicated by the SNR algorithm are shown.

**Figure 11:**
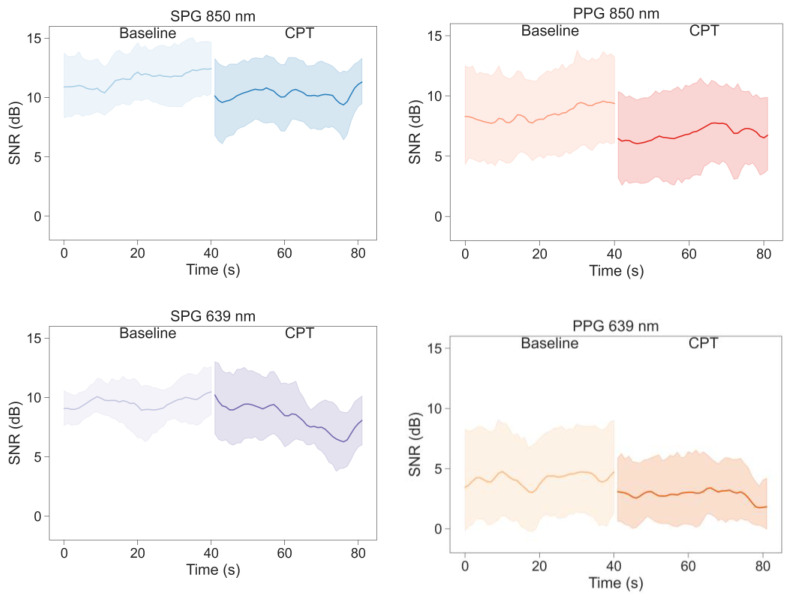
Average values and 95% CI of the SNR of the signals extracted from the videos during baseline and CPT.

Figure 11 shows average values and 95% CI of the SNR values inter-subject for the 4 signals produced by the experimental device (PPG and SPG at 850 nm and 639 nm) differentiating between the two phases of the study (baseline and CPT). Most signals show a reduction in SNR during the CPT phase, except for SPG 639 nm, which has a less abrupt SNR change between the two study phases.

**Figure 12:**
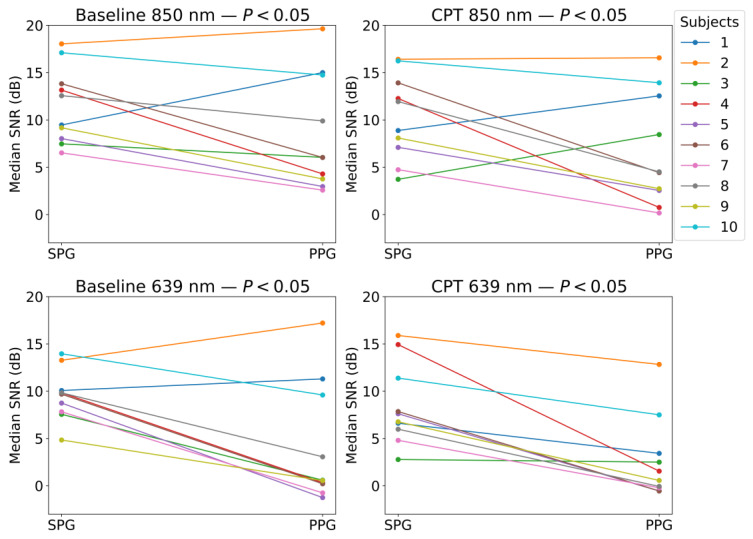
All-subject overview of SPG and PPG SNR values at both wavelengths for baseline and CPT phases. *p*-values shown on paired Wilcoxon test, reveal significance of null-hypothesis rejection, showing that SNR SPG is significantly higher than SNR PPG.

As shown in Figure 11, the SNR is higher for SPG than for PPG at all wavelengths and phases. Figure 11 further confirms this by showing the comparison between the SNR of SPG and PPG at two different wavelengths differentiating between the different phases of the study. Wilcoxon signed-rank test results, indicated in the titles of Figure 12, show that for both wavelengths and phases, the SNR of SPG is significantly higher than the SNR of PPG.

Examination of Figure 11 and Figure 12 reveals a large spread in the SNR between subjects for PPG compared to SPG. Confirmed by Levene’s test, a significant difference in variance was observed between SPG and PPG during baseline at 850 and 639 nm and during the CPT only at 850 nm.

**Figure 13:**
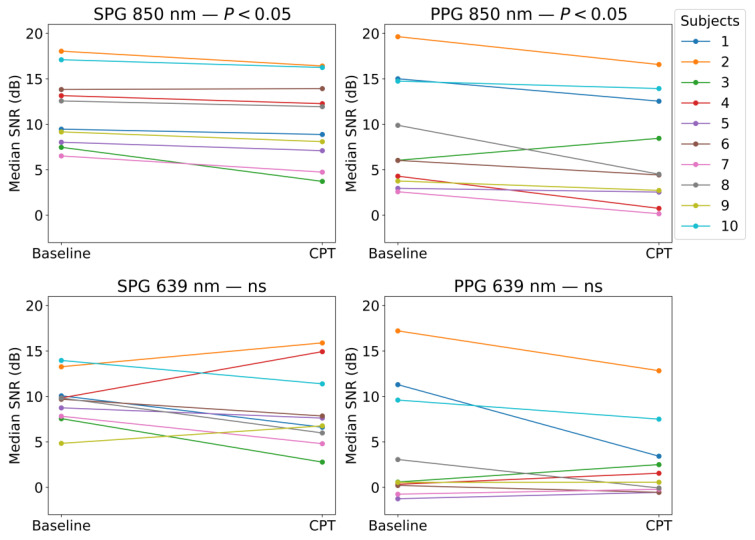
Median SNR values on baseline and CPT phases shown for every subject on each of the experimental signals. Wilcoxon test *p*-values smaller than 0.05 show the significance of the rejection of the null-hypothesis; this indicates that the SNR baseline is significantly higher than the SNR CPT. The term “ns” refers to non-significant.

An overview on the reduction in the SNR due to the CPT for every experimental signal on every subject is presented in Figure 11. The significance of the Wilcoxon signed-rank test is presented in the titles of Figure 13.

Similar to the results for the AC-amplitude analysis, at 850 nm, both SPG and PPG showed a significant reduction in the SNR during the CPT case, and at 639 nm, the difference that resulted is not significant.

### 3.3. Harmonic Ratio Results

**Figure 14:**
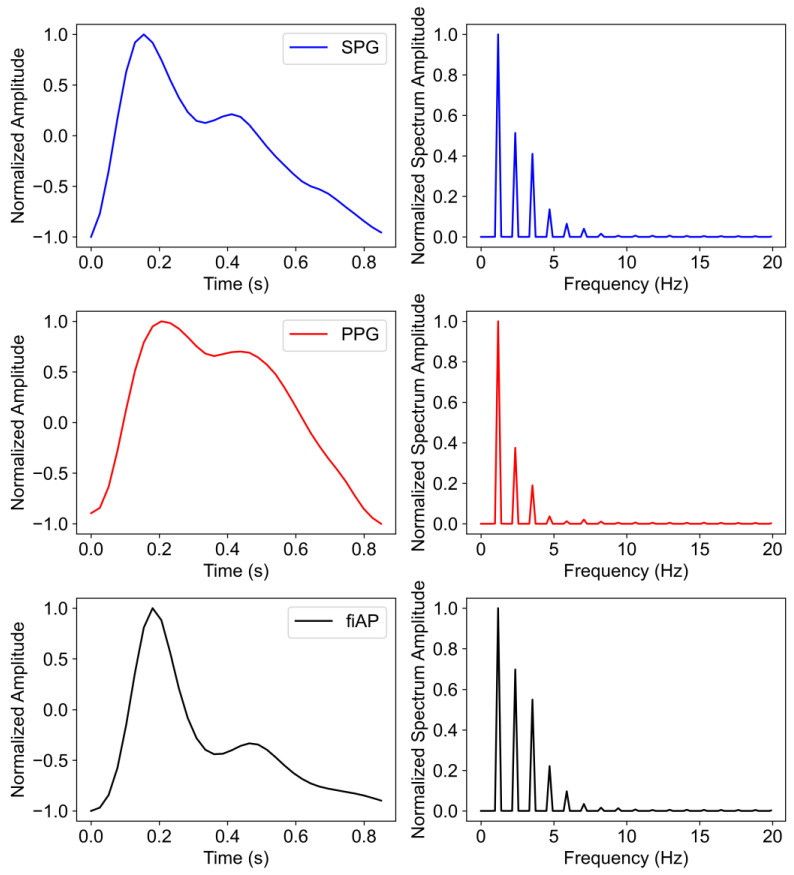
Representative beat measured from subject 10 (**left**) and its Fourier spectrum (**right**) from experimental (SPG and PPG) and reference (fiAP) signals during baseline. Note, the beats were band-passed filtered to remove low frequency drift, respiration influence, and high frequency noise, and normalized to improve comparability.

The harmonic ratios, SHR, THR, and FHR were analysed to highlight the morphology differences between 850 nm SPG, 850 nm PPG, and fiAP representative beats from each subject during the baseline phase of the study. Figure 14 shows an example of the representative SPG, PPG, and fiAP beats or heart cycles (left) with their calculated Fourier spectrum (right).

**Figure 15:**
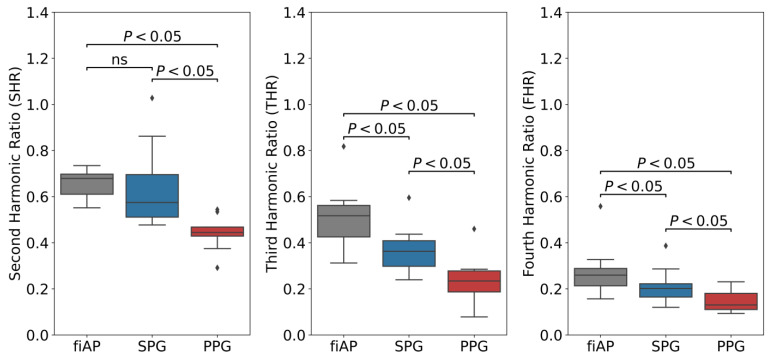
Boxplot of harmonic ratios of fiAP, SPG, and PPG during baseline including *p*-values from Wilcoxon test (*p* < 0.05 means significantly higher harmonic ratio between of the signal on the left of the bracket compared to the one on the right). All displayed SPG and PPG values were measured at 850 nm. The term “ns” refers to non-significant.

The boxplots of the SHR, THR, and FHR calculated for the representative beats are presented in Figure 15 together with the results of the Wilcoxon signed-rank test. In particular, all the harmonic ratios of PPG resulted in being significantly lower than the SPG and fiAP ones. Furthermore, while SPG’s THR and FHR were significantly lower than the ones of fiAP, the same comparison for SHR was not significant.

**Figure 16:**
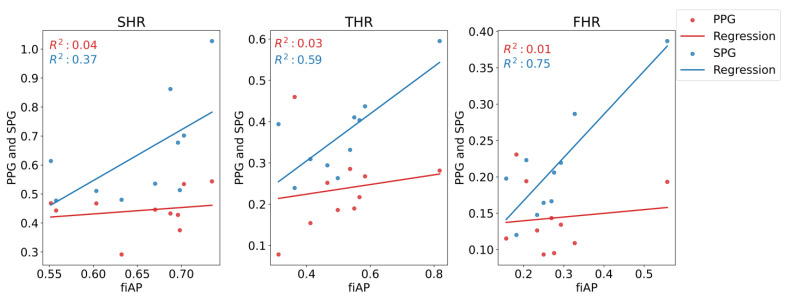
Correlation plots of the harmonic ratios between the fiAP reference and PPG and SPG. The regression lines are also shown, and the coefficient of determination is indicated. All displayed SPG and PPG values were measured at 850 nm.

In Figure 16, the correlation plots between each harmonic ratio of fiAP and PPG/SPG is shown. The correlation between fiAP and SPG is significantly higher than the one between fiAP and PPG for all harmonic ratios.

## 4. Discussion

In this work, reflective PPG and SPG were obtained at two wavelengths (639 and 850 nm, using time-multiplexing) from the right index finger of 10 healthy volunteers, using fiAP as a reference under lowered perfusion conditions by applying a CPT. The CPT consisted of submerging the subject’s hand in ice water for 1 min. This commonly causes cold-induced vasoconstriction, leading to lowered perfusion conditions also in the non-immersed hand where the measurements took place. The data collection consisted of a baseline phase and a CPT phase. Per wavelength, PPG and SPG were calculated from the same video stream using different processing pipelines.

As seen in the Results Section, both AC-amplitude and SNR of PPG and SPG decrease during CPT-induced lowered perfusion conditions. This only reached statistical significance for the signals at a wavelength of 850 nm and not for 639 nm. Furthermore, SPG has consistently higher SNR values than PPG, with lower intra- and inter-subject variation. In addition, the harmonic analyses revealed that there is a larger resemblance between SPG and fiAP compared to PPG and fiAP.

In healthy subjects, the CPT leads to an increase in BP and a decrease in blood flow and volume. It is expected that PPG will have a reduced AC-amplitude as the blood volume decreases. Since SPG is a speckle-based method, it might be influenced by blood flow [1] as well as by blood pressure [8]. From the AC-amplitude analysis, it is shown that the change due to the CPT was significant with 850 nm illumination (see Figure 9). Both SPG and PPG signals at 850 nm showed a significant decrease, while both signals at 639 nm did not show a significant decrease. This could suggest that the signals at the two wavelengths partly contain different information. A possible explanation might be the difference in penetration depth or changes in chromophores [19]. However, the non-significance of the AC-amplitude decrease in PPG at 639 nm could also be caused by the lower quality, and hence variation, of the signal overall. fiAP, on the other hand did not show a significant reduction as expected. The PP showed an increase during the CPT, compared to the baseline, of 9% ± 10%.

SPG resulted in having a significantly higher SNR compared to PPG in both the baseline and CPT, and both wavelengths, as shown in Figure 11 and Figure 12. During a CPT, the reduction in volumetric flow in the peripheral circulation and the shunting of the capillaries influences the SNR of both PPG and SPG. These lower perfusion conditions depict a challenging situation for PPG measurements. However, the SNR of SPG is significantly higher than PPG during normal conditions but also during the CPT. Therefore, in poor perfusion cases where PPG could be lost, SPG might still preserve enough signal quality for heart rate (HR) measurements. In addition, the variance in the SNR was found to be significantly different between SPG and PPG for all phases and light sources applied, with one exception. This exception was PPG at 639 nm during the CPT, where the SNR was already low before the CPT and became consistently low across subjects during the CPT, reaching a consistency in SNR levels similar to the SPG. This explains how the SNR across subjects during the two phases of the measurement is significantly more consistent for SPG.

The change in SNR was further analysed for each signal between the two phases, as shown in Figure 13. Similar to the AC-amplitude changes, for 850 nm illumination, there was a statistically significant difference in the SNRs between the baseline and CPT phases. This means that both PPG and SPG have a statistically significant reduction in the SNR during the CPT. This reduction is less present at 639 nm, however, it is worth mentioning that the SNR for PPG at 639 nm was already very poor during baseline measurements, as shown in Figure 11. This might explain the fact that the decrease in the SNR is not significant.

These results indicate that SPG could be more accurate than PPG for HR measurements for most situations, and especially in conditions with low perfusion. Furthermore, this could even extend to more accurate Heart Rate Variability (HRV) and Pulse Arrival Time (PAT) measurements over long recording periods [8,20], which could be applied for more accurate non-invasive blood pressure assessment.

In all subjects, the harmonic ratios measured during baseline on the 850 nm illumination source were found to be significantly different between SPG and PPG, as visible in Figure 15. fiAP shows the most pronounced harmonic ratios, closely followed by SPG, and PPG shows the lowest harmonic ratios. This means that the secondary peak is usually more pronounced for fiAP and SPG than for PPG. SHR was not significantly different between fiAP and SPG. The correlation between fiAP and SPG is higher than PPG for all harmonic ratios (see Figure 16), which suggests a bigger relation of SPG with BP than PPG. It could be speculated that differences between harmonic ratios derived from simultaneous SPG and PPG signals might provide information about the state of the circulatory system, but this requires more investigation. However, considering the differences in SPG morphology depend on age, as already shown in [1], this analysis should be repeated on a larger population. SPG has potential to be used as a method to assess cuffless blood pressure, but care should be taken to directly use AC values, since they are still subjected to perfusion changes independent of BP.

This is the first investigation analysing dual-wavelength SPG and PPG from a reflective contact probe. Measuring in reflective mode is suitable for more body locations than transmission mode; it allows for more compact setups and offers the possibility of integration in wearable devices. In addition, the custom setup designed for this work adds extra information due to the dual-wavelength usage. However, the relatively small population, 10 subjects, forms a limitation of the study. This is especially relevant if the differences in CPT reactions between different ages and sexes are considered [21].

## 5. Conclusions

The CPT has an effect on the SNR of both SPG and PPG signals; at 850 nm, most of the participants show a decrease in the SNR under the CPT. However, the SNR of SPG was significantly higher than the PPG one, with significantly lower variability. This suggests that SPG measurements could be more suitable for low perfusion situations. The AC-amplitude of SPG and PPG signals at 850 nm show a significant reduction during the CPT, but this is not seen for 639 nm. This suggests that the two wavelengths might contain different information and might be affected differently by haemodynamics.

The harmonic ratio analysis indicates that fiAP, PPG and SPG are different signals, containing different information about the state of the cardiovascular system and blood dynamics. Harmonic ratio analysis revealed clear differences between SPG and PPG. Furthermore, the correlation between fiAP and SPG is significantly higher than the one between fiAP and PPG.

Thus, to provide more information on the monitoring of blood dynamics, combining SPG and PPG measurements should be considered, instead of using only PPG. Furthermore, while low perfusion conditions that can compromise the pulse amplitude are challenging to monitor with PPG, SPG might offer better results. Some examples of such conditions are Raynaud’s syndrome, diabetes, vasculitis, and advanced age. Studies on a larger population, including healthy subjects and patients with such conditions, should be conducted to further conclude on these points.

## Data Availability

The data used in this study are not publicly available due to privacy concerns.
